# The 2023 South Sudanese outbreak of Hepatitis E emphasizes ongoing circulation of genotype 1 in North, Central, and East Africa

**DOI:** 10.1016/j.meegid.2024.105667

**Published:** 2024-10

**Authors:** Gregory S. Orf, Nicholas Bbosa, Michael G. Berg, Robert Downing, Sonja L. Weiss, Deogratius Ssemwanga, Alfred Ssekagiri, Shirin Ashraf, Ana da Silva Filipe, Ronald Kiiza, Joshua Buule, Hamidah Suubi Namagembe, Stella Esther Nabirye, John Kayiwa, Lul Lojok Deng, Gregory Wani, James A. Maror, Andrew Baguma, Juma J.H. Mogga, Saleem Kamili, Emma C. Thomson, Pontiano Kaleebu, Gavin A. Cloherty

**Affiliations:** a Core Diagnostics, Abbott Laboratories, Abbott Park, IL, USA; b Abbott Pandemic Defense Coalition, Abbott Park, IL, USA; c Uganda Virus Research Institute, Entebbe, Uganda; d MRC/UVRI & LSHTM Uganda Research Unit, Entebbe, Uganda; e MRC-University of Glasgow Centre for Virus Research, Glasgow, Scotland, UK; f National Public Health Laboratory (NPHL), Ministry of Health, Juba, South Sudan; g World Health Organization, Juba, South Sudan; h Department of Microbiology, Kabale University School of Medicine, Kabale, Uganda; i Epidemiology and Surveillance Branch, U.S. Centers for Disease Control, Atlanta, GA, USA; j Queen Elizabeth University Hospital, Glasgow, Scotland, UK

**Keywords:** Hepatitis E virus, Acute hepatitis, South Sudan, Internally displaced people, Outbreak, Next-generation sequencing

## Abstract

In April 2023, an outbreak of acute hepatitis was reported amongst internally displaced persons in the Nazareth community of South Sudan. IgM serology-based screening suggested the likely etiologic agent to be Hepatitis E virus (HEV). In this study, plasma specimens collected from anti-HEV IgM-positive cases were subjected to additional RT-qPCR testing and sequencing of extracted nucleic acids, resulting in the recovery of five full and eight partial HEV genomes. Maximum likelihood phylogenetic reconstruction confirmed the genomes belong to HEV genotype 1. Using distance-based methods, we show that genotype 1 is best split into three sub-genotypes instead of the previously proposed seven, and that these sub-genotypes are geographically restricted. The South Sudanese sequences confidently cluster within sub-genotype 1e, endemic to northeast, central, and east Africa. Bayesian Inference of phylogeny incorporating sampling dates shows that this new outbreak is not directly descended from other recent local outbreaks for which sequence data is available. However, the analysis suggests that sub-genotype 1e has been consistently and cryptically circulating locally for at least the past half century and that the known outbreaks are often not directly descended from one another. The ongoing presence of HEV, combined with poor sanitation and hygiene in the conflict-affected areas in the region, place vulnerable populations at risk for infection and its more serious effects, including progression to fulminant hepatitis.

## Highlights

• In April 2023, an outbreak of acute hepatitis caused by Hepatitis E virus (HEV) was reported amongst internally displaced persons in South Sudan.
• Next-generation sequencing of patient plasma yielded five full and eight partial HEV genomes which belong to genotype 1, sub-genotype 1e.
• Phylogenetic analyses suggest that there are three true sub-genotypes within genotype 1, and that these sub-genotypes are geographically restricted.
• Temporal analysis suggests that sub-genotype 1e has been cryptically circulating in North, Central, and East Africa for the past half century.
• We stress the need for to acquire more full-length HEV genomes to enhance surveillance efforts and improve molecular epidemiology studies.

## 1 Introduction

Hepatitis E virus (HEV; species *Paslahepevirus balayani*) is a major cause of acute viral hepatitis worldwide and infects a wide variety of mammals (Kamar et al., 2017). Eight major genotypes of HEV have been identified, with four of these (genotypes 1–4) regularly infecting humans (Smith et al., 2020). Genotypes 1 and 2 are associated with fecal-oral transmission between humans, whereas genotypes 3 and 4 are transmitted to humans in a zoonotic fashion from ungulates (*e.g.*, swine) or rodents (*e.g.*, rabbits) (Doceul et al., 2016; Kamar et al., 2014). While infection with HEV is typically mild and resolves on its own within 4–6 weeks, it can progress to fulminant hepatitis and jaundice, impacting liver function and causing death in 0.5–4 % of individuals (Kamar et al., 2014; Disease Outbreak News, 2023). Individuals who are pregnant, immunocompromised, or living with pre-existing liver disease are at greater risk for mortality (Marano et al., 2015).

Following civil war and unrest during the 2010's, internally displaced people (IDP) in South Sudanese refugee settlements currently face poor sanitation and hygiene, exacerbated by over-crowding, periodic flooding, and a scarcity of clean water (South Sudan: Regional Refugee Response Plan, 2022). As a result, outbreaks of HEV transmitted primarily through contaminated drinking water have become a regular occurrence. For example, in the Bentiu IDP Settlement of Unity state, South Sudan, where over 100,000 IDP live, there have been over 4000 cases reported through early 2023, resulting in 27 deaths (Disease Outbreak News, 2023). Outside of South Sudan, outbreaks in Sudan have been consistently reported over the last decade (Disease Outbreak News, 2021), and sporadic reports from Egypt (Sayed and Abdelwahab, 2022), Kenya (Ahmed et al., 2013), Central African Republic (De Marguerite Nombot-Yazenguet et al., 2024; Tricou et al., 2020), Cameroon (Modiyinji et al., 2021), and Uganda (Teshale et al., 2010; Gerbi et al., 2015) speak to the persistence of HEV in North, Central, and East Africa.

A new outbreak of HEV was recently declared by the South Sudan Ministry of Health on April 14, 2023 (Disease Outbreak News, 2023). Beginning in late March there were 91 suspected cases amongst IDPs in the Nazareth community of the city of Wau, with 35 of these confirmed by anti-HEV IgM testing. Though the outbreak unfortunately resulted in 5 deaths, a joint response team composed of the Ministry of Health and World Health Organization mobilized to identify routes of exposure and halt the spread. To verify the serology testing results from this outbreak, 24 plasma specimens were transported to neighboring Uganda for qPCR-based molecular testing, with 10 of these returning positive results.

Here, we describe whole-genome sequencing results from a subset of 24 plasma specimens collected during the recent Nazareth IDP outbreak. We analyzed these genomes in the context of others collected in the region, including new partial genomes from an earlier 2008 outbreak in Uganda and a 2012 outbreak in Ugandan and South Sudan. We utilize Genetic Distance, Maximum Likelihood, and Bayesian Inference methods, to assign sub-genotypes to the new sequences and ascertain their phylogenetic histories.

## 2 Methods

### 2.1 Serological and molecular testing

The National Public Health Laboratory (NPHL) of South Sudan received 24 specimens for testing, between 23 March 2023 and 09 April 2023, from patients in Wau with suspected Acute Jaundice Syndrome. All 24 tested negative for Rift Valley Fever Virus and Yellow Fever Virus by RT-qPCR. Of these, 15 specimens were further tested for HEV using the ASSURE HEV IgM Rapid Test (MP Biomedicals, Eschwege, Germany; tests donated by Médecins Sans Frontières), with 14 returning positive results.

Remaining volumes of all 24 specimens were shipped to the Uganda Virus Research Institute (UVRI), a collaborating regional laboratory, for HEV-specific RT-qPCR testing. Upon delivery to the UVRI on 13 April 2023, total nucleic acid (TNA) was extracted from 240 μl of each specimen using the semi-automated NucliSens easyMAG platform (bioMérieux SA, Marcy-l'Étoile, France), with a final elution volume of 50 μl. Two microliters of TNA from each specimen and from positive controls were amplified using an HEV-specific RT-qPCR probe-hydrolysis assay (Jothikumar et al., 2006) performed on a QuantStudio 7 Pro Real-Time PCR System (ThermoFisher Scientific, Waltham, USA).

### 2.2 Next-generation sequencing

Sequencing of the outbreak specimens was also performed at the UVRI. After unbiased reverse transcription of the RNA component of extracted TNA, unbiased (metagenomic) sequencing libraries were constructed from DNA/cDNA using the Nextera DNA Prep (formerly Nextera DNA Flex) library preparation kit and DNA/RNA Unique Dual Indexes (Illumina, San Diego, USA). Aliquots of each metagenomic library were also enriched for viral sequences using the Viral Surveillance Panel (VSP; Illumina, San Diego, USA), which contains specific probes for Hepatitis E virus and others listed as important public health risks by the WHO. Libraries were sequenced on a MiSeq instrument using a MiSeq Reagent Kit v3 and 2 × 150 bp sequencing chemistry (Illumina, San Diego, USA).

### 2.3 Bioinformatic analysis

FASTQ files containing sequencing results were uploaded to Abbott's “DiVir3” pipeline for bioinformatic analysis, which identifies both known and divergent pathogen reads. Read hits of interest were further investigated by *de novo* contig assembly and/or iterative mapping against reference sequences using CLC Genomics Workbench v.23 (Qiagen Corp. Germantown, MD, USA). To confirm a virus identification, we implemented a variation of established criteria stating that at least three distinct regions of its genome must be covered by at least one high-quality paired-end sequencing read, which should generally result in at least 4 % of the genome being recovered (Miller et al., 2019; Mourik et al., 2024; Schlaberg et al., 2017). Consensus genomes and coverage statistics were collated and exported for further analysis. An in-house implementation of the BLAST algorithm (Altschul et al., 1990) and MAFFT v.7.487 aligner (Katoh and Standley, 2013) were also utilized for sequence comparison.

### 2.4 Sequence dataset construction

**Fig. 1:**
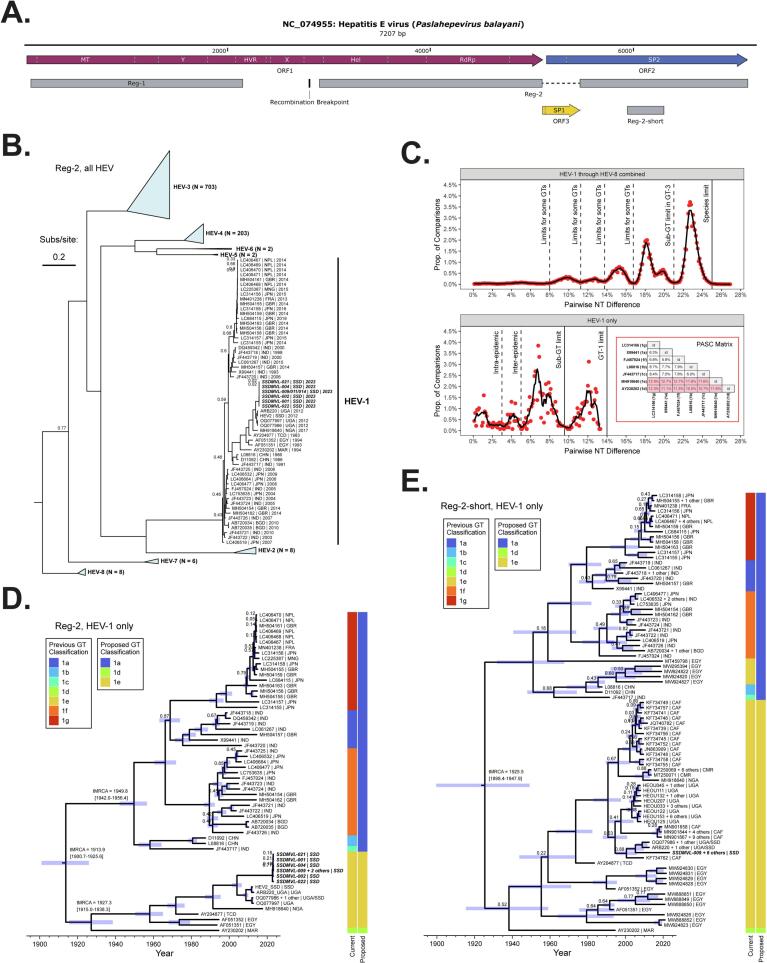
Phylogenetic reconstruction of HEV, with an emphasis on genotype 1, using Maximum Likelihood, Genetic Distance, and Bayesian Inference methods. A. Genomic map of HEV annotated with genes, functional domains, and phylogenetic markers (MT: methyltransferase; Y: protease; HVR: hypervariable region; X: macro domain; Hel: helicase; RdRp: RNA-dependent RNA polymerase; SP: structural protein). B. ML tree calculated from an MSA of Reg-2, with the sequences introduced in this study in bold italic font. Genotypes other than HEV-1 are collapsed for clarity, with the number of sequences constituting each subtype denoted. The tree is rooted using Moose HEV (*Paslahepevirus alci*) as an outgroup (taxon not shown). Scale bars for the branch lengths are shown in units of substitutions per site. Any Ultrafast Bootstrap supports <0.9 are explicitly shown at nodes. The taxon labels follow the format “Accession Number (or Specimen Number) | Collection Country (three letter standard abbreviation) | Collection Year”. C. Frequency distribution of pairwise distances computed from 821 HEV sequences (top) and 53 HEV genotype 1 sequences (bottom) using the PASC method. Dots represent raw data and the black lines represent a smoothed line generated using the Savitzky-Golay filter (Savitzky and Golay, 1964). Appropriate proposed cut-off values for genetic distance differentiating genotypes and sub-genotypes are noted. D. and E. Bayesian MCC trees inferred from MSAs of Reg-2 and Reg-2-short, respectively, for HEV genotype 1, incorporating sampling date. The 95 % highest probability density (HPD) for height is shown at ancestral nodes as a semi-transparent blue bar, with some time-of-most-recent-common-ancestor (tMRCA) values (plus 95 % HPD) highlighted. Any posterior probabilities <0.9 are explicitly shown at nodes. To the right of each tree is a color-coded key describing the current and proposed sub-genotype assignments; the proposed sub-genotype assignments are based on the genotype 1 distance cut-offs described in panel C. Abbreviations: GT – genotype. (For interpretation of the references to color in this figure legend, the reader is referred to the web version of this article.)

All known complete or nearly complete genome sequences of Hepatitis E virus (*Paslahepevirus balayani*) were extracted from the GenBank and ViPR databases (identified using a combination of BLAST and taxonomy searching) and placed into a dataset containing our genomes from the 2023 South Sudan outbreak. Sequences known to have numerous recombination breakpoints (*e.g.*, (Luk et al., 2018)) were omitted. Genomes determined by NGS from the 2012 HEV outbreaks in South Sudan (sequence names HEV1/HEV2) and Uganda (Ashraf et al., 2023) (sequence name ARB220) were communicated to us by Dr. Robert Downing and Dr. Emma C. Thomson, respectively, and added to the dataset. Sequences without a collection date or location noted in the metadata were manually investigated in the literature; any sequences still lacking these metadata were discarded. Remaining sequences were de-duplicated such that the earliest-collected sequence in any identical set was retained. The full genomes were aligned using the L-INS-i algorithm of MAFFT v.7.487 (Katoh and Standley, 2013) to produce a multiple sequence alignment (MSA). Using guidance from a previous study by Forni et al. (Forni et al., 2018), the MSA was split into two recombination-free regions, Reg-1 and Reg-2, with repetitive domains and overlapping coding regions masked. Reg-1 (∼2080 nt) contains the N-terminus of ORF1. Due to its longer length and antigenicity, focus was placed on Reg-2 (∼3850 nt), which contained the helicase and polymerase domains of ORF1 and the regions of ORF2 (structural protein) that do not overlap with ORF3 (Fig. 1A). Finally, sequence entries in each region with more than 10 % ambiguous or missing nucleotides were discarded. To increase the entries belonging to HEV genotype 1e, we also included outbreak sequences from Egypt, Central African Republic (CAR), Cameroon, Sudan, and Uganda which only had a short (∼360 nt) region within Reg-2 sequenced (denoted Reg-2-short). Reg-2-short sequences determined *via* Sanger sequencing from the 2008–2009 HEV outbreak (Teshale et al., 2010) in Uganda (sequence names HEOU033, HEOU034, HEOU035, HEOU036, HEOU045, HEOU088, HEOU111, HEOU122, HEOU125, HEOU132, HEOU134, HEOU153, HEOU181, HEOU182, HEOU191, HEOU193, HEOU194, HEOU207, HEOU222) were communicated to us by Dr. S. Kamili and added to this dataset. Thus, three datasets were considered for phylogenetic analysis: 1) all sequences from all genotypes of HEV spanning Reg-2's complete length (992 sequences), 2) a subset of dataset 1 containing only HEV genotype 1 (59 sequences), and 3) dataset 2, clipped to Reg-2-short, including additional HEV genotype 1e sequences spanning Reg-2-short (85 sequences).

### 2.5 Maximum likelihood reconstruction of phylogeny

The evolutionary history of Dataset 1, focusing on recapitulating the delineation of HEV genotypes, was reconstructed using the maximum likelihood (ML) method as deployed in IQTREE v.2.1.3 (Minh et al., 2020). Briefly, the ModelFinder algorithm (Kalyaanamoorthy et al., 2017) was used to find a suitable nucleotide substitution model (here, GTR+F+R10) using Bayesian Information Criterion as the scoring method. Initial ML tree reconstruction was achieved using a stochastic algorithm and then optimized using the Nearest Neighbor Interchange (Robinson, 1971; Moore et al., 1973) heuristic method. The tree with the best log-likelihood score (the ML tree) was retained, and branch supports were calculated using 1000 replicates of Ultrafast Bootstrapping (Hoang et al., 2018).

### 2.6 Bayesian inference of phylogeny

The evolutionary history of Datasets 2 and 3, focusing on the emergence dates of various regional epidemics and subtypes of HEV genotype 1, was reconstructed using Bayesian Inference (BI). First, the presence of temporal signal in each dataset was confirmed: ML trees for datasets 2 and 3 were constructed like above, and TempEst v.1.5.3 (Rambaut et al., 2016) was used to plot sampling date *versus* root-to-tip distance; linear regression suggested good clock signal with a substitution rate of roughly 1 × 10^−3^ subs site^−1^ yr^−1^ with R^2^ value >0.6 and no discernable outliers. To perform BI reconstruction of phylogeny, datasets 2 and 3 were analyzed with BEAST v.1.10.4 (Suchard et al., 2018) using the Markov Chain Monte Carlo (MCMC) method with a General Time Reversible (GTR) substitution model with empirical base frequencies and gamma distribution with 4 categories (the same models chosen by ModelFinder during ML reconstruction). A strict clock model and constant size coalescent tree prior (constant population size prior with exponential distribution and mean of 10), suggested by Forni et al. (Forni et al., 2018), were used, although Bayesian Estimation of Temporal Signal (BETS) (Duchene et al., 2020) and model selection/marginal likelihood estimation using path sampling and stepping-stone sampling (Baele et al., 2012) were also used to confirm temporal signal and determine the validity of an alternative clock (uncorrelated relaxed lognormal clock) and alternative tree prior (exponential growth) (see Supplementary Information, Tables S1-S2 and Fig. S1). MCMC analyses were run with 5 × 10^7^ chains, sampled every 5 × 10^3^ chains. Runs were checked for convergence and suitable ESS values (> 200) for each variable using Tracer 1.7.2 (Rambaut et al., 2018). Maximum clade credibility (MCC) trees were generated after removal of 10 % burn-in using TreeAnnotator v.1.10.4 (Suchard et al., 2018). All phylogenetic trees were visualized and annotated with metadata using the *ggtree* and *ggplot2* packages (Yu et al., 2017; Wickham, 2016) for the *R* v.4.3.1 programming language.

### 2.7 Genotype and sub-genotype classification

MSAs of Datasets 1 and 2 (all HEV and HEV genotype 1, respectively, over the complete Reg-2 marker) were analyzed using the method of Pairwise Sequence Comparison (PASC) (Bao et al., 2008). Sequence identity matrices were generated using BioEdit v.7.7.1. The matrices were then processed using a custom script, written in the *R* programming language, to compute and plot the prevalence of individual pairwise identities within the alignments. Genotypic and sub-genotypic classifications were made based on the cut-offs observed in the plots and cross-referenced back to the phylogenetic trees.

## 3 Results

### 3.1 An outbreak of HEV was detected in South Sudan during May 2023

Upon determination that the etiologic agent of an acute jaundice/hepatitis outbreak was likely to be HEV, twenty-four plasma specimens were sent to the reference laboratory at the Uganda Virus Research Institute (UVRI) for confirmatory testing and sequencing (Table 1). At UVRI, molecular testing by RT-qPCR revealed that 10 of the 24 specimens (∼42 %) had detectable levels of HEV ribonucleic acid. Leftover plasma volumes (available for 23 of the 24 specimens) were then processed for metagenomic and virus-enriched NGS on the Illumina platform. Fourteen of the 23 sequenced specimens (61 %) passed our internal criteria for positive pathogen identification (Materials and Methods) and returned ≥9 % genome coverage: over 98 % coverage was recovered for five specimens (identifying numbers ending in −002, −009, −011, −014, and − 021), while between 9 and 97 % coverage was recovered for nine more (identifying numbers ending in −001, −003, −004, −008, −012, −013, −015, −022, and − 024). Of the RT-qPCR positive specimens, 8/10 (80 %) returned genomes with ≥87 % coverage and 2/10 (20 %) returned genomes with between 9 and 49 % coverage. Of the RT-qPCR negative specimens that were sequenced (13/14 specimens), 4/13 (31 %) returned genomes with ≥20 % coverage.

**Table 1:** Diagnostic testing and next-generation sequencing results for the twenty-four specimens collected during the 2023 South Sudan HEV outbreak at the Nazareth IDP Settlement and processed at the UVRI. Confidence in HEV assignment by sequencing was defined by genome recovery level (≥98 %: high; 11–97 %: medium; 4–10 %: low); however, at least three distinct genomic regions required coverage to pass the minimum criteria for “low” confidence. Cov: coverage; Dx: diagnostic.

Specimen ID	Dx testing	Next-generation sequencing				
	RT-qPCR	HEV reads	HEV cov. (%)	HEV depth (X)	HEV confidence	Other pathogens detected
A23–04-001-SSDMLV-001	+	3750	87	63.5	Medium	none
A23–04-002-SSDMLV-002	+	63,186	100	1117.8	High	*Plasmodium falciparum*
A23–04-003-SSDMLV-003	−	838	47	15.8	Medium	none
A23–04-004-SSDMLV-004	+	17,696	95	303.1	Medium	Measles virus, SARS-CoV-2
A23–04-005-SSDMLV-005	−	6	3	0	None	none
A23–04-006-SSDMLV-006	−	0	0	0	None	none
A23–04-007-SSDMLV-007	−	6	3	0	None	none
A23–04-008-SSDMLV-008	+	1471	49	27.1	Medium	none
A23–04-009-SSDMLV-009	+	35,463	98	637.2	High	HIV-1
A23–04-010-SSDMLV-010	−	8	7	0.1	None	none
A23–04-011-SSDMLV-011	+	237,435	100	4179.8	High	none
A23–04-012-SSDMLV-012	−	132	28	2.6	Medium	none
A23–04-013-SSDMLV-013	−	199	15	3.9	Medium	none
A23–04-014-SSDMLV-014	+	15,481	98	262.9	High	none
A23–04-015-SSDMLV-015	−	261	20	4.6	Medium	none
A23–04-016-SSDMLV-016	−	5	0	0	None	HIV-1, *Rickettsia felis*
A23–04-017-SSDMLV-017	−	4	2	0	None	HIV-1, SARS-CoV-2
A23–04-018-SSDMLV-018	−	4	0	0	None	none
A23–04-019-SSDMLV-019	−	6	7	0.1	None	none
A23–04-020-SSDMLV-020	−	*insufficient volume for sequencing*				
A23–04-021-SSDMLV-021	+	62,883	98	1126.7	High	none
A23–04-022-SSDMLV-022	+	15,841	90	295.4	Medium	none
A23–04-023-SSDMLV-023	−	0	0	0	None	none
A23–04-024-SSDMLV-024	+	274	9	4.4	Low	none

Five specimens appeared to have co-infections: one with HEV and malaria (*Plasmodium falciparum*), one with HEV, measles virus, and SARS-CoV-2, one with HEV and HIV-1, one with HIV-1 and *Rickettsia felis*, and one with HIV-1 and SARS-CoV-2. Specimens A23–04-016-SSDMLV-016 and A23–04-017-SSDMLV-017, which contained HIV-1, appeared to contain other opportunistic pathogens as well, such as *Trichobilharzia spp.* and *Aspergillus spp.* The three HIV-1 genomes recovered had only 25–40 % genome coverage but had >90 % nucleotide identity to each other in shared mapped regions. The closest BLAST hits to the HIV-1 genomes were subtype A strains from Kenya and Uganda. Mapping statistics for these coinfections may be found in the Supplementary Information, Table S3.

### 3.2 HEV strains from the April 2023 South Sudan outbreak belong to the Africa-centric clade of genotype 1

To obtain insight into the evolutionary history of the complete or mostly complete HEV genomes recovered in this study, we combined them with currently available full-genome references from the viral species *Paslahepevirus balayani* (regardless of host). We were also able to acquire three extra genomes from a previous Ugandan and South Sudanese outbreak from 2012 that are not currently available in GenBank (specimen numbers HEV1/HEV2/ARB220). With these sequences incorporated, we reconstructed an ML tree using the recombination-free (Forni et al., 2018) phylogenetic marker Reg-2 (Fig. 1A and B). The branching patterns of the major genotypes (1 through 8) were well-supported by Ultrafast Bootstrapping and consistent with other studies (Doceul et al., 2016; Forni et al., 2018). In Reg-2, all 2023 South Sudanese sequences had >99.7 % identity to one another and all 2012 Ugandan/South Sudanese sequences had >99.1 % identity to one another. These outbreaks collectively formed a well-supported monophyletic group with a sequence from the 1983 outbreak amongst French soldiers in Chad (van Cuyck-Gandre et al., 1997), a sequence from the 2017 outbreak in Nigeria (Akanbi et al., 2019), and three sequences from Egypt and Morocco from the early 1990's (Tsarev et al., 1999; Chen and Meng, 2004), firmly within HEV genotype 1, hereafter referred to as HEV-1. The Chadian and Nigerian sequences have been previously classified as a distinct sub-genotype, HEV-1e, thus we initially assigned the 2023 South Sudanese sequences to this sub-genotype as well.

### 3.3 The current classification of sub-genotypes within HEV-1 is unsupported by molecular evidence

To better understand the diversity within HEV broadly, and within HEV-1 more specifically, we performed a distance-based analysis called Pairwise Sequence Comparison (PASC; Fig. 1C). This method allows for the statistical derivation of cut-off values for genetic distance that conform to taxonomic delineations such as genus or species. When full genomes of all known strains belonging to the formalized *Paslahepevirus balayani* species are considered (Fig. 1C, top panel) the most important cut-off observed is at 25 % nucleotide difference; thus, essentially no two strains within this species display more than 25 % nucleotide difference between them. However, many more cut-offs corresponding to genotype and sub-genotype delineations exist, specifically at 21, 17, 14, 11.5, and 8 % nucleotide differences. Mapping these differences back to the ML tree in Fig. 1B, reveals a non-uniform application of the definition of genotype and sub-genotype based on common nucleotide difference criteria. This trend has been noted before (Smith et al., 2020).

However, when sequences annotated as HEV-1 are isolated into their own alignment and the PASC analysis is repeated (Fig. 1C, bottom panel), a clear nucleotide difference limit of 14 % is observed, meaning that all HEV-1 sequences share at least 86 % nucleotide identity. The next nucleotide difference cut-off observed is at ∼9.5 %; this appears to correspond best to sub-genotype delineations, meaning that strains within the same sub-genotype should have nucleotide differences of less than 9.5 %. Sub-genotype designations recently proposed by Smith and coworkers (Smith et al., 2020) include 7 entries, 1a through 1 g; however, our proposed nucleotide difference limit of 9.5 % does not support the existence of this many sub-genotypes. Reference sequences for proposed sub-genotypes 1a, 1b, 1c, 1f, and 1 g are all <8.4 % different from each other and are all >10.5 % different from the reference sequences for proposed sub-genotypes 1d and 1e (Fig. 1C, bottom panel inset). Additionally, the reference sequences for genotypes 1d and 1e are 11.6 % different from each other. Thus, it is appropriate to reclassify sub-genotypes 1a, 1b, 1c, 1f, and 1 g as a single unit: sub-genotype 1a. Furthermore, when these delineations are mapped onto a phylogenetic tree inferred using sampling dates (*i.e.*, through Bayesian Inference, Fig. 1D and E), the reduced sub-genotype list of 1a, 1d, and 1e neatly segregate by geographic region: 1a in Asia, 1d in northwest Africa (Morocco), and 1e in northeast, central, and east Africa.

### 3.4 HEV genotype 1 likely emerged in the early 1900's and differentiated into the current sub-genotypes before 1960

A time-resolved maximum clade credibility (MCC) tree derived through Bayesian Inference, including all HEV genotype 1 sequences with sufficient coverage in the Reg-2 marker (Fig. 1D), reveals a most recent common ancestor (MRCA) of all HEV-1 to have emerged around the year 1914 (median decimal year: 1913.9; 95 % highest posterior density [HPD]: 1900.7–1925.6). In this tree's topology (unlike that of the ML tree), the clade containing African strains and the clade containing Asian strains split at the root. Chronologically, the next branching event occurred around the year 1927 (median decimal year: 1927.3; 95 %-HPD: 1915.0–1938.3); this event represents a split between sub-genotype 1d identified in Morocco and sub-genotype 1e found in Egypt and Central/East Africa. Within the Asian clade, the chronologically first branching event represents a split between what has previously been defined as sub-genotypes 1b/1c and what has previously been defined as sub-genotypes 1a/1c/1 g (median decimal year: 1949.8; 95 %-HPD: 1942.0–1956.4). As indicated in the previous section, when our proposed sub-genotype classification is applied to the topology of this tree, all Asian strains belong to a unified sub-genotype 1a, the northwest African strain (accession AY230202) solely belongs to sub-genotype 1d, and all Egyptian and Central/East African strains belong to sub-genotype 1e. The emergence time of the MRCA of all proposed extant genotype 1a sequences is calculated to be in the year 1949 (median: 1949.8, 95 %-HPD: 1942.0–1956.4).

Bayesian Inference of phylogeny was repeated with an abbreviated sub-region of the Reg-2 marker (Reg-2-short), utilizing additional African strains previously collected through short-amplicon Sanger sequencing (Fig. 1E). Though posterior probabilities for many of the major early branching events are below 0.9, the general topology of this MCC tree is largely the same as that generated for Reg-2 (Fig. 1D). The geographic separation amongst proposed genotypes is maintained in this tree, except for the appearance of partial genomes of recent Egyptian strains (accessions MT459798, MW294820, MW294822, MW294827, and MW295394) within the major clade containing all Asian sequences. Despite the inability to integrate these Egyptian sequences into the PASC analysis due to their short size, their position in the tree suggests that they belong to our proposed unified sub-genotype 1a, as opposed to 1e like other Egyptian strains (*e.g.*, accessions AF051351 and AF051352).

### 3.5 The MRCA of HEV sub-genotype 1e likely emerged in the 1960's and has caused sporadic outbreaks ever since

The MCC trees for both Reg-2 and Reg-2-short markers (Fig. 1D and E) suggest an emergence date for the most recent common ancestor of all extant genotype 1e sequences to be around 1960 (median: 1958.2; 95 %-HPD: 1950.3–1964.8, and median: 1959.0; 95 %-HDP: 1943.2–1972.4, respectively). According to both trees, this date corresponded to a split between Egyptian and non-Egyptian (*i.e.*, Central/East African) lineages. Despite the absence of genomic coverage in the Reg-1 marker for the foundational Egyptian sequences of sub-genotype 1e (*i.e.*, accessions AF051351 and AF051352), we calculated an MCC tree for this marker anyway (Supplementary Information, Fig. S2). While the general topology resolved for sub-genotype 1e (and its split from the other sub-genotypes) is the same, the date corresponding to its emergence is delayed by roughly a decade (median: 1967.2; 95 %-HPD: 1960.1–1973.6). Due to the more complete nature of sampling in the Reg-2 and Reg-2-short markers for our sub-genotype of interest, we maintain our focus on those markers.

Chronologically, the first sub-genotype 1e sequence recovered from the non-Egyptian lineage was the 1983 outbreak amongst French soldiers stationed in Chad. In the tree for Reg-2 (Fig. 1D), the next branching event (occurring in roughly 1991) represents the split between a lineage containing the 2017 Nigerian outbreak and a lineage containing the 2012 Uganda/South Sudan and 2023 South Sudan outbreaks. The tree also suggests that none of the sampled sequences from the 2012 Uganda/South Sudan outbreak are the direct ancestor of the sampled sequences from the 2023 outbreak; these share a MRCA that emerged in 2004 (median: 2004.1; 95 %-HPD: 2001.5–2006.5). Despite these new insights, utilizing this longer phylogenetic marker prevents resolving the placement of numerous other outbreaks occurring between 1993 and 2023 in the region.

The MCC tree inferred using the Reg-2-short marker (Fig. 1E) reveals that the sporadic outbreaks in CAR (2008–2009 and 2018–2019), Uganda (2008–2009, 2010–2012), Cameroon (2013), Nigeria (2017), South Sudan (2012), and South Sudan (2023) all share a MRCA with an emergence date around 1991, recapitulating the equivalent emergence date calculated using the Ugandan, South Sudanese, and Nigerian sequences alone in Reg-2 (Fig. 1D). There are various important observations to be made in this second MCC tree when considering the relatedness of one outbreak to the next within the same country. First, it appears that the 2018–2019 CAR outbreak is not closely related to, or descended from, the 2008–2009 CAR outbreak; these share a MRCA (∼1991) with all other known outbreaks in the region. However, it also appears that the 2008–2009 outbreak did not consist of a single source or lineage; the sequence from GenBank accession KF734762 is more closely related to the recent South Sudanese outbreaks than it is to the 13 other local sequences collected contemporaneously with it. Similarly, the 2008–2009 Ugandan outbreak is not closely related to the 2012 Ugandan/South Sudan outbreak; these also last share an MRCA with the other known outbreaks in the region. Third, it appears that the closest sampled relative to both the 2012 Ugandan/South Sudan outbreak and 2023 South Sudan outbreak is the aforementioned accession KF734762 from CAR; this sequence is not the direct ancestor of these outbreaks but shares a MRCA with them that is inferred to have emerged in 2000 (median: 2000.6; 95 % HPD: 1994.7–2005.6). This tree also confirms the results from Fig. 1D showing that the 2023 South Sudan outbreak is not directly descended from the 2012 outbreak. Fourth, the 2013 Cameroonian outbreak appears closely related to the 2017 Nigerian outbreak; they share a MRCA that appears to have emerged in roughly 2009. Lastly, we note the genetic diversity in the sequences recently collected from sporadic outbreaks in Egypt: some appear to be close relatives of sequences first identified in 1993–1994, while others cluster more confidently near the root of sub-genotype 1a.

## 4 Discussion

It is estimated that in the decade spanning 2010–2020, at least 12 independent HEV outbreaks occurred amongst IDP in sub-Saharan Africa, totaling >30,000 cases and > 600 deaths (Desai et al., 2022). Even outside of IDP or refugee camps, poor sanitation, recurrent flooding, undeveloped infrastructure, and overcrowding contribute to two types of epidemiological trends: a) large, sporadic HEV outbreaks which may be poorly or belatedly investigated (Kim et al., 2014), and b) low-level, but consistent, circulation that goes mostly undiagnosed (Sayed and Abdelwahab, 2022). Whole genome surveillance of HEV has not kept pace with the growing number of outbreaks. Indeed, in our study, we encountered numerous fragmentary genomes (<400 bp in length) deposited to online databases that hinder more advanced molecular epidemiology study. To achieve a better reconstruction of sub-genotype 1e and provide health authorities with additional relevant information, we utilized next-generation sequencing to recover as many full HEV genomes as possible from the April 2023 South Sudan outbreak.

The newly collected genomes expand the known genetic diversity within HEV-1, and their inclusion in our PASC analysis firmly corroborates the existence of the distinct sub-genotype 1e geographically constrained to North, Central, and East Africa. The analysis also shows that the 2023 South Sudan outbreak is not the direct descendent of previous outbreaks in Uganda/South Sudan (2012) and nearby CAR (2008–2009) and Uganda (2008–2009); in fact, these outbreaks share inferred common ancestors that emerged in 2006, 2002, and 1993, respectively. Furthermore, the MCC tree generated from the Reg-2-short marker suggests an expansion of the Central/East African lineages of sub-genotype 1e in the 1990's that eventually gave rise to at least 8 major outbreaks. Bayesian Skyline reconstruction of genetic diversity, particularly if more complete genomes are recovered, could provide more insight into any such expansion. However, these current lines of evidence, together with the known 1983 outbreak in Chad, speak to established, endemic, cryptic circulation of HEV-1e in the region for at least the past half century. While some European HEV-1a strains have been identified (Davis et al., 2021; Marion et al., 2020), presumably imported by travelers from Asia, we did not find evidence of the import of non-African lineages in our South Sudan cohort.

The branching patterns observed in the MCC trees for both phylogenetic marker regions (Fig. 1D, E, and Supplementary Information, Fig. S2) demonstrate that African strains, particularly the Moroccan sub-genotype 1d sequence, lie closest to the root of HEV-1. We postulate that early HEV-1 diversification events were associated with a geography switch from northwest Africa (Morocco) to northeast Africa (Egypt), or *vice-versa*, which resulted in the original splits between sub-genotypes 1d, 1e, and 1a. Furthermore, new Egyptian sequences collected in the past four years (*e.g.*, accession MW295394) appear near the root of our proposed unified sub-genotype 1a, indicating a possible original northeast African origin for the Asian strains of sub-genotypes 1a. These lines of evidence together imply that the most recent common ancestor of all HEV-1 may have originally emerged in Africa after splitting from the sister lineage that would become HEV-2. Combined with a recent increase in complete HEV-2 genomes available from Senegal (Sadio et al., 2022) and Nigeria (Wang et al., 2018) and older partial genomes available from Nigeria (Buisson et al., 2000), Chad (Nicand et al., 2005), and Namibia (Maila et al., 2004), it is becoming apparent that Bayesian phylogeographic reconstructions should be repeated to better establish the ancestral location of both HEV-1 and HEV-2. Incorporating these recent high-quality sequences (including our South Sudanian full genomes) may well demonstrate an African origin for the last common ancestor of these two genotypes, as opposed to an Asian origin as proposed recently (Forni et al., 2018).

Amongst the 20 countries with the most conflict-driven IDP (IDMC, 2024), about one-third lie within the geographic range of HEV sub-genotype 1e. Outbreak preparedness and response should be a shared responsibility where health authorities work together with non-profits, academia, and the private sector, transcending international borders (Averhoff et al., 2022). Therefore, we encourage those scientific and medical staff working in resource-limited locations, such as IDP camps, to collaborate in a multisector, networked approach to obtain as much diagnostic and genomic information as possible when a new outbreak arises, even of a known pathogen. High importance should be placed on acquiring full genomes of the pathogen in question (Davis et al., 2021), so that molecular epidemiology studies can be undertaken to afford useful information on outbreak source or new mutations that may affect disease severity or immune escape.

## Ethics statement

The study was approved by the Uganda Virus Research Institute Research Ethics Committee (UVRI-REC), approval GC/127/908, and the Uganda National Council of Science and Technology (UNCST), approval HS2543ES.

## Funding

Funding supporting laboratory work and analysis (*i.e.*, HEV-specific RT-qPCR, sequencing, and phylogenetics) was provided by Abbott Laboratories as part of the Abbott Pandemic Defense Coalition program; this funder also provided support in the form of salaries for the authors employed by Abbott Laboratories but did not have any additional role in the study design, data collection and analysis, decision to publish, or preparation of the manuscript. Additional funding supporting field and laboratory work (*i.e.*, serology and other RT-qPCR testing) was provided by the World Health Organization, Africa CDC, and Médecins Sans Frontières. Funding supporting field logistics was provided by the Ministry of Health of Uganda.

## CRediT authorship contribution statement

**Gregory S. Orf:** Writing – review & editing, Writing – original draft, Visualization, Validation, Software, Methodology, Investigation, Formal analysis, Conceptualization. **Nicholas Bbosa:** Writing – review & editing, Supervision, Project administration, Methodology, Investigation, Conceptualization. **Michael G. Berg:** Writing – review & editing, Supervision, Project administration, Conceptualization. **Robert Downing:** Supervision, Investigation. **Sonja L. Weiss:** Writing – review & editing, Validation, Investigation, Data curation. **Deogratius Ssemwanga:** Writing – review & editing, Supervision, Resources. **Alfred Ssekagiri:** Writing – review & editing, Investigation. **Shirin Ashraf:** Writing – review & editing, Investigation. **Ana da Silva Filipe:** Writing – review & editing, Investigation. **Ronald Kiiza:** Writing – review & editing, Investigation. **Joshua Buule:** Writing – review & editing, Resources. **Hamidah Suubi Namagembe:** Writing – review & editing, Investigation. **Stella Esther Nabirye:** Writing – review & editing, Investigation. **John Kayiwa:** Writing – review & editing, Resources. **Lul Lojok Deng:** Writing – review & editing, Investigation. **Gregory Wani:** Writing – review & editing, Investigation. **James A. Maror:** Writing – review & editing, Investigation. **Andrew Baguma:** Writing – review & editing, Investigation. **Juma J.H. Mogga:** Writing – review & editing, Investigation. **Saleem Kamili:** Writing – review & editing, Resources. **Emma C. Thomson:** Writing – review & editing, Supervision. **Pontiano Kaleebu:** Writing – review & editing, Supervision, Funding acquisition. **Gavin A. Cloherty:** Writing – review & editing, Supervision, Funding acquisition.

## Declaration of competing interest

GSO, SLW, MGB, and GAC are all employees and shareholders of Abbott Laboratories. The remaining authors declare that the research was conducted in the absence of any commercial or financial relationships that could be construed as a potential conflict of interest.

## Data Availability

The new sequencing data presented in this article (i.e., HEV genomes from the May 2023 South Sudan outbreak with over 40 % coverage) are available at the NCBI GenBank repository with accession numbers PQ268479-PQ268488. Scripts, nucleotide alignments, or BEAST/phylogenetic tree files will be made available upon reasonable request to the corresponding author.

## Appendix A Supplementary data

Supplementary material, including in-depth explanation of phylogenetics methods, Tables S1-S3, and Figures S1-S2. Image 1
