# Supplementary material for: The 2023 South Sudanese outbreak of Hepatitis E emphasizes ongoing circulation of genotype 1 in North, Central, and East Africa
Source: Infect Genet Evol. 2024 Oct;124:105667. doi: 10.1016/j.meegid.2024.105667 (PMC11413618; doi:10.1016/j.meegid.2024.105667)
Supplement: Supplementary file 1 — Supplementary material, including in-depth explanation of phylogenetics methods, Tables S1-S3, and Figures S1-S2. [file mmc1.docx]

**Supplementary Material for:**

**The 2023 South Sudanese outbreak of Hepatitis E emphasizes ongoing circulation of genotype 1 in North, Central, and East Africa**

Gregory S. Orf^1,2,*^, Nicholas Bbosa^2,3,4^, Michael G. Berg^1,2^, Robert Downing^2,3^, Sonja L. Weiss^1,2^, Deogratius Ssemwanga^3,4^, Alfred Ssekagiri^3^, Shirin Ashraf^5^, Ana da Silva Filipe^5^, Ronald Kiiza^4^, Joshua Buule^3^, Hamidah Suubi Namagembe^4^, Stella Esther Nabirye^3^, John Kayiwa^3^, Lul Lojok Deng^6^, Gregory Wani^6^, James A. Maror^6^, Andrew Baguma^7,8^, Juma J.H. Mogga^7^, Saleem Kamili^9^, Emma C. Thomson^5,10^, Pontiano Kaleebu^3,4^, Gavin A. Cloherty^1,2^

^1^Core Diagnostics, Abbott Laboratories, A­­­­­­­­­bbott Park, IL, USA

^2^Abbott Pandemic Defense Coalition, Abbott Park, IL, USA­­­

^3^Uganda Virus Research Institute, Entebbe, Uganda

^4^MRC/UVRI & LSHTM Uganda Research Unit, Entebbe, Uganda

^5^MRC-University of Glasgow Centre for Virus Research, Glasgow, Scotland, UK

^6^National Public Health Laboratory (NPHL), Ministry of Health, Juba, South Sudan

^7^World Health Organization, Juba, South Sudan

^8^Department of Microbiology, Kabale University School of Medicine, Kabale, Uganda

^9^Epidemiology and Surveillance Branch, U.S. Centers for Disease Control, Atlanta, GA, USA

^10^Queen Elizabeth University Hospital, Glasgow, Scotland, UK

^*^To whom correspondence should be addressed: [gregory.orf@abbott.com](mailto:gregory.orf@abbott.com)

# Supplementary Methods

Bayesian Evaluation of Temporal Signal (BETS) (1) was utilized as an alternative method for establishing the presence of temporal signal in our datasets, as implemented in BEAST v.1.10.4 (2). BETS compares the log marginal likelihood estimation (MLE) calculated from an MCMC run using contemporaneous tip sampling (isochronous data) against the log MLE calculated from a second MCMC run using dated tip sampling (heterochronous data); the comparison of these two log MLEs is called the log Bayes Factor (BF). BETS was performed on the alignments of both datasets (*i.e.*, Reg-2 and Reg-2-short) using a strict clock and constant size coalescent tree prior (constant population size prior with exponential distribution and mean of 10). MCMC analyses were run with 5 ×10^7^ chains, sampled every 5 ×10^3^ chains. Runs were checked for convergence and suitable ESS values (> 200) for each variable (after 10% burn-in) using Tracer 1.7.2 (3). MLE was performed after each run using both path sampling and stepping-stone sampling (4-6), with 100 path steps each with chain length of 5 ×10^5^ (resulting in the same number of total chains, 5 ×10^7^, as the MCMC analysis).

Using a similar methodology as BETS, one additional molecular clock model (uncorrelated relaxed clock with lognormal distribution; UCLD) and one additional tree prior (exponential population growth; EG) were tested using comparison of log MLE. In this case, only dated tip sampling (heterochronous model) was used. Thus, four total MCMC analyses (one for each clock model with each tree prior) were conducted per dataset.

# Supplementary Results

## Phylogenetic Model Selection

Root-to-tip analysis of our maximum likelihood phylogenies was performed using TempEst v.1.5.3 (7), suggesting that a hypothesis of a strict molecular clock underlying the evolution within our dataset was well-supported, with substitution rates ~1 ×10^-3^ subs site^-1^ yr^-1^, with R^2^ > 0.6 and no discernable outliers. A secondary evaluation of temporal signal using a strict clock hypothesis and Bayesian methods, also known as BETS, was performed. BETS showed clear preference for the heterochronous model (tip dating) in each dataset, with log BF >> 20 (**Table S1**), suggesting that further dated analyses were warranted. For the dataset containing Reg-2, a median clock rate of 1.168 ×10^-3^ (95% HPD: 1.021 ×10^-3^ – 1.329 ×10^-3^) substitutions/site/year was calculated, whereas for Reg-2-short, a median clock rate of 1.580 ×10^-3^ (95% HPD: 1.156 ×10^-3^ – 2.104 ×10^-3^) substitutions/site/year was calculated.

**Table S1: Bayesian Evaluation of Temporal Signal (BETS).** Using a strict clock and constant size population tree prior (each contain a limited number of assumptions), BETS was performed as a second evaluation of the "clocklikeness" of each dataset. The log Bayes factor (BF) is computed as the difference between the log marginal likelihood estimation (MLE) obtained from using two different models: contemporaneous tip sampling (isochronous) versus dated tip sampling (heterochronous) (BF = MLE_1_ − MLE_2_; if positive, model 1 is favored). BF > 20 is considered significant, though BF > 150 is most desirable (8).

| **Dataset** | Reg-2 | | Reg-2-short | |
| --- | --- | --- | --- | --- |
| **Clock** | Strict | | Strict | |
| **Tree Prior** | Constant Size | | Constant Size | |
| **Sampling** | Path | Stepping-Stone | Path | Stepping-Stone |
| **log MLE, isochronous** | -21,892.62 | -21,891.65 | -3,485.34 | -3,485.49 |
| **log MLE, heterochronous** | -21,761.03 | -21,761.91 | -3,438.51 | -3,439.77 |
| **log BF (hetero − iso)** | 131.59 | 129.74 | 46.83 | 45.72 |
| **Temporal signal?** | Yes | Yes | Yes | Yes |

Though recent work (9) has suggested the use of a strict molecular clock assumption, we wished to test an alternative hypothesis, the uncorrelated relaxed local clock with lognormal distribution (UCLD). Aside from a constant size (CS) coalescent tree prior (which contains minimal assumptions), we also wished to test the more complicated exponential growth tree prior (EG) as well (**Table S2**). Using the log BF comparison, the UCLD was favored over SC for the Reg-2 marker, but not for the Reg-2-short marker. Additionally, neither the CS or EG tree prior was favored over the other, thus we used the CS prior due to its simplicity and use of fewer assumptions.

**Table S2: Model selection using path sampling and stepping-stone sampling.** Two clock models were tested using a constant size population tree prior: a strict clock (SC) and uncorrelated relaxed clock with lognormal distribution (UCLD). The best clock model was then tested against two tree priors: constant size (CS) population and exponential growth (EG) population. The log Bayes Factor (BF) is computed in the same way as in **Table S1** with the same interpretations (*e.g.*, BF > 20 is considered significant).

| **Dataset** | Reg-2 | | Reg-2-short | |
| --- | --- | --- | --- | --- |
| **Independent variable** | Clock | | Clock | |
| **Tree Prior** | Constant Size | | Constant Size | |
| **Sampling** | Path | Stepping-Stone | Path | Stepping-Stone |
| **log MLE, SC** | -21,761.03 | -21,761.91 | -3,438.51 | -3,439.77 |
| **log MLE, UCLD** | -21,719.36 | -21,719.43 | -3,438.21 | -3,439.40 |
| **log BF (UCLD − SC)** | 41.66 | 42.49 | 0.30 | 0.37 |
| **Favored** | UCLD | UCLD | neither | neither |
|  |  |  |  |  |
| **Dataset** | Reg-2 | | Reg-2-short | |
| **Independent variable** | Tree Prior | | Tree Prior | |
| **Clock** | UCLD | | SC (fewer assumptions) | |
| **Sampling** | Path | Stepping-Stone | Path | Stepping-Stone |
| **log MLE, CS** | -21719.36 | -21719.43 | -3438.51 | -3439.77 |
| **log MLE, EG** | -21722.74 | -21723.29 | -3434.85 | -3435.84 |
| **log BF (EG − CS)** | -3.38 | -3.87 | 3.66 | 3.93 |
| **Favored** | neither | neither | neither | neither |
|  |  |  |  |  |
| **Dataset** | Reg-2 | | Reg-2-short | |
| **Best clock** | UCLD | | SC | |
| **Best tree prior** | CS | | CS | |

Though the UCLD clock was favored over the SC clock for the Reg-2 marker (log BF ~ 42, which is greater than the log BF ~ 20 threshold for significance), a maximum clade credibility (MCC) tree generated from the corresponding MCMC analysis indicated little local variability in the evolutionary rate within the Africa-centric clade of HEV-1, with the rate ranging from 1.192 ×10^-3^ to 1.676 ×10^-3^ substitutions/site/year (**Figure S1**). In contrast, the rate varies more in the Asia-centric clade of HEV-1, ranging from 0.474 ×10^-3^ to 2.694 ×10^-3^ substitutions/site/year. Additionally, the topologies of the two MCC trees (inferred using the SC vs UCLD) are identical in the Africa-centric clade. While in the main text, we show the MCC tree for Reg-2 using the SC hypothesis due to its more simplistic nature, here in this Supplement we also present the tree generated for the Reg-2 maker using the UCLD hypothesis for the sake of completeness.


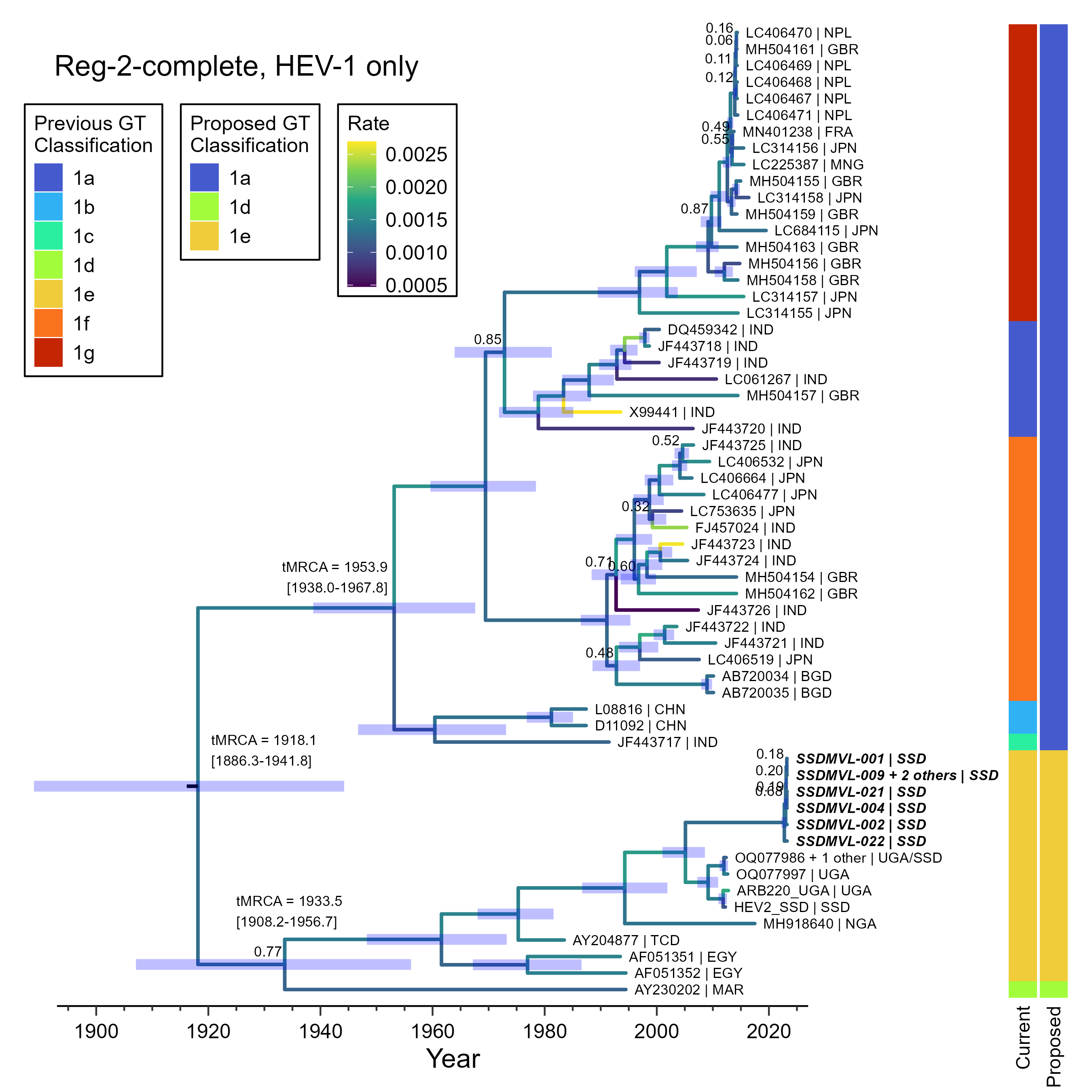


**Figure S1: Tip-dated MCC tree inferred for the Reg-2 phylogenetic marker using an UCLD clock hypothesis.** The 95% highest probability density (HPD) for height is shown as blue bars at ancestral nodes, with some time-of-most-recent-common-ancestor (tMRCA) values (plus 95% HPD) highlighted. Branches are color-coded according to the calculated local molecular clock rate. Any posterior probabilities <0.9 are explicitly shown at nodes. The taxon labels are in the following format: “Accession or strain number | three-letter standard code for collection country.” To the right of the tree is a color-coded key describing the current and proposed sub-genotype assignments; the proposed assignments are based on the genotype 1 distance cut-offs described in the Main Text, Figure 1, panel C.

## Detection of Co-infections

As described in the **Main Text**, five of the analyzed specimens appeared to have co-infections: one with HEV and malaria (*Plasmodium falciparum*), one with HEV, measles virus, and SARS-CoV-2, one with HEV and HIV-1, one with HIV-1 and *Rickettsia felis*, and one with HIV-1 and SARS-CoV-2. Below (**Table S3**) is an accounting of NGS mapping statistics for those specimens. Despite the low read count for the non-viruses *Plasmodium falciparum* and *Rickettsia felis*, the read mappings do span at least three regions of the genome and are high-quality reads; therefore, we putatively assign them as low confidence positives despite not reaching 4% reference coverage as would be required for viruses.

**Table S3: Next-generation sequencing results for the five specimens collected during the 2023 South Sudan HEV outbreak at the Nazareth IDP Settlement with suspected coinfections.** Confidence in virus assignment by sequencing was defined by genome recovery level (≥98%: high; 11-97%: medium; 4-10%: low); however, at least three distinct genomic regions required coverage to pass the minimum criteria for “low” confidence. cov: coverage.

| Specimen ID | **Next-generation sequencing** | | | | |
| --- | --- | --- | --- | --- | --- |
|  | **Pathogen** | **Reads** | **Genome cov. (%)** | **Cov. depth (X)** | **Confidence** |
| A23-04-002-SSDMLV-002 | HEV | 63,186 | 100 | 1,117.8 | High |
| A23-04-002-SSDMLV-002 | *Plasmodium falciparum* | 23 | < 1 | < 0.01 | Low |
| A23-04-004-SSDMLV-004 | HEV | 17,696 | 95 | 303.1 | Medium |
| A23-04-004-SSDMLV-004 | Measles virus | 101 | 13 | 0.8 | Medium |
| A23-04-004-SSDMLV-004 | SARS-CoV-2 | 3,650,547 | 100 | 15,514.7 | High |
| A23-04-009-SSDMLV-009 | HEV | 35,463 | 98 | 637.2 | High |
| A23-04-009-SSDMLV-009 | HIV-1 | 13,137 | 27 | 194.4 | Medium |
| A23-04-016-SSDMLV-016 | HIV-1 | 36,527 | 25 | 492.0 | Medium |
| A23-04-016-SSDMLV-016 | *Rickettsia felis* | 6 | < 1 | < 0.01 | Low |
| A23-04-017-SSDMLV-017 | HIV-1 | 32,148 | 40 | 463.8 | Medium |
| A23-04-017-SSDMLV-017 | SARS-CoV-2 | 1,795,873 | 96 | 6,834.1 | High |

## Concordance of topologies between trees generated from the Reg-1 and Reg-2 phylogenetic markers

A major disadvantage of relying on the Reg-1 phylogenetic marker, denoted in the **Main Text, Figure 1A**, is the fact that there is no coverage in this region for two of the foundational sub-genotype 1e genomes, namely those collected from Egypt in the 1990’s (accession numbers AF051351 and AF051352). Despite this, we reconstructed a time-dependent phylogeny for this marker using the same priors and strict clock as for the Reg-2 marker (depicted in the **Main Text, Figure 1D**). The median evolutionary rate of the strict clock was determined to be 1.24 ×10^-3^ subs/site/yr (95%-HPD: 1.03 ×10^-3^ to 1.46 ×10^-3^ subs/site/yr), comparable to that determined for the Reg-2 marker (median: 1.17 ×10^-3^ subs/site/yr; 95%-HPD: 1.02 ×10^-3^ to 1.33 ×10^-3^ subs/site/yr).

The MCC tree generated for the Reg-1 marker (**Figure S2**) shows a nearly identical overall topology of that generated for the Reg-2 marker (**Main Text, Figure 1D**) with good statistical support; namely, an early split between sub-genotypes 1a and 1d/1e, with a subsequent split of sub-genotypes 1d and 1e shortly thereafter. The inferred dates of these splits are roughly the same as well across the two phylogenetic markers. However, due to the lack of the foundational Egyptian sequences in the Reg-1 marker dataset, the inferred emergence date of the last common ancestor of all extant sampled sub-genotype 1e sequences (median: 1967.2; 95%-HPD: 1960.1-1973.6) does differ from that inferred from the Reg-2 marker dataset (median: 1958.2; 95%-HPD: 1950.3-1964.8) by nearly a decade.


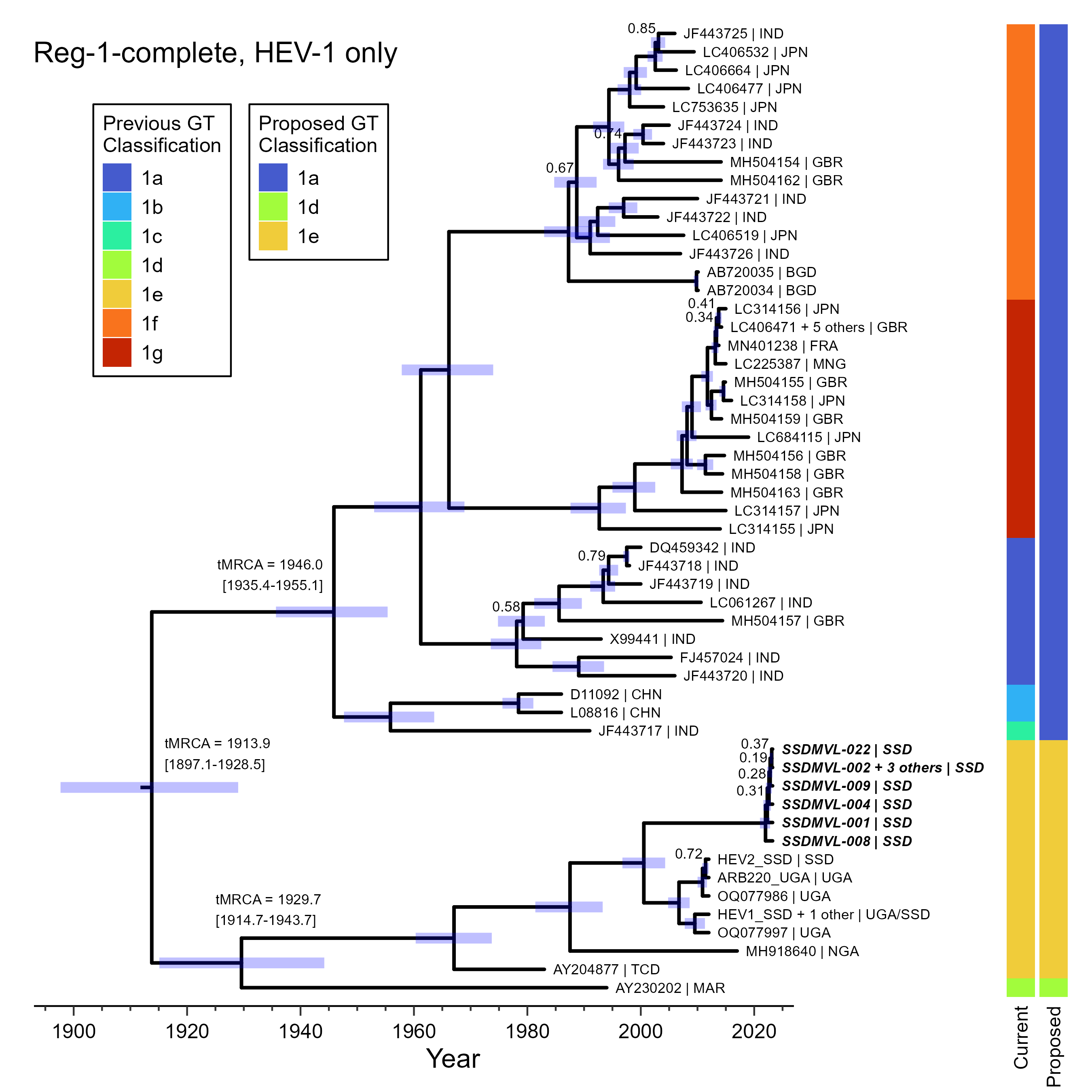


**Figure S2: Tip-dated MCC tree inferred for the Reg-1 phylogenetic marker using a strict clock hypothesis.** The tree is inferred from an MSA of Reg-1 for HEV genotype 1, incorporating sampling date. The 95% highest probability density (HPD) for height is shown at ancestral nodes as a semi-transparent blue bar, with some time-of-most-recent-common-ancestor (tMRCA) values (plus 95% HPD) highlighted. Any posterior probabilities <0.9 are explicitly shown at nodes. To the right of the tree is a color-coded key describing the current and proposed sub-genotype assignments; the proposed sub-genotype assignments are based on the genotype 1 distance cut-offs described in the Main Text, Fig. 1, panel C. Abbreviations: GT – genotype.

# Supplementary References

1. Duchene S, Lemey P, Stadler T, Ho SYW, Duchene DA, Dhanasekaran V, Baele G. Bayesian Evaluation of Temporal Signal in Measurably Evolving Populations. Mol Biol Evol. 2020 Nov 1;37(11):3363-3379. Epub 2020/09/09. doi:10.1093/molbev/msaa163. Cited in: Pubmed; PMID 32895707.

2. Suchard MA, Lemey P, Baele G, Ayres DL, Drummond AJ, Rambaut A. Bayesian phylogenetic and phylodynamic data integration using BEAST 1.10. Virus Evolution. 2018 2018-01-01;4(1). doi:10.1093/ve/vey016.

3. Rambaut A, Drummond AJ, Xie D, Baele G, Suchard MA. Posterior Summarization in Bayesian Phylogenetics Using Tracer 1.7. Syst Biol. 2018 Sep 1;67(5):901-904. Epub 2018/05/03. doi:10.1093/sysbio/syy032. Cited in: Pubmed; PMID 29718447.

4. Baele G, Lemey P, Bedford T, Rambaut A, Suchard MA, Alekseyenko AV. Improving the Accuracy of Demographic and Molecular Clock Model Comparison While Accommodating Phylogenetic Uncertainty. Molecular Biology and Evolution. 2012 2012-09-01;29(9):2157-2167. doi:10.1093/molbev/mss084.

5. Lartillot N, Philippe H. Computing Bayes Factors Using Thermodynamic Integration. Systematic Biology. 2006 2006-04-01;55(2):195-207. doi:10.1080/10635150500433722.

6. Xie W, Lewis PO, Fan Y, Kuo L, Chen M-H. Improving Marginal Likelihood Estimation for Bayesian Phylogenetic Model Selection. Systematic Biology. 2011 2011-03-01;60(2):150-160. doi:10.1093/sysbio/syq085.

7. Rambaut A, Lam TT, Max Carvalho L, Pybus OG. Exploring the temporal structure of heterochronous sequences using TempEst (formerly Path-O-Gen). Virus Evol. 2016 Jan;2(1):vew007. Epub 2016/10/25. doi:10.1093/ve/vew007. Cited in: Pubmed; PMID 27774300.

8. Kass RE, Raftery AE. Bayes Factors. Journal of the American Statistical Association. 1993;(430):773-795. Epub 2012-02-27.

9. Forni D, Cagliani R, Clerici M, Sironi M. Origin and dispersal of Hepatitis E virus. Emerging Microbes & Infections. 2018 2018-12-01;7(1):1-13. doi:10.1038/s41426-017-0009-6.
